# A Method to Track Targets in Three-Dimensional Space Using an Imaging Sonar

**DOI:** 10.3390/s18071992

**Published:** 2018-06-21

**Authors:** Danxiang Jing, Jun Han, Jin Zhang

**Affiliations:** 1 Institute of Marine Information Engineering, Zhejiang University, Zhoushan 316021, China; jingdxiang@zju.edu.cn; 2 College of Marine Sciences, Shanghai Ocean University, Shanghai 201306, China; j_zhang@shou.edu.cn

**Keywords:** underwater positioning, data association, 3D tracking, ARIS

## Abstract

This paper introduces a methodology applying an imaging sonar for three-dimensional (3D) target tracking underwater. The key process in this work involves obtaining the target’s position in space using two images of the same scene, acquired by an adaptive resolution imaging sonar (ARIS) at different positions. A data association algorithm was designed to connect the same target in image sequences. The goal of this work was to track multiple targets in 3D space. The ARIS provides sequences of bi-dimensional images from the backscattered energy according to the range and azimuth. The challenge involved determining the missing elevation information for the observed object within the sonar detection range. By computing the geometrical transformation between the acquisition planar images and the cubical space, using only the sonar information that included the posture and moving speed of the ARIS, the target’s elevation information was obtained. To evaluate the performance of the proposed method, an indoor experiment was conducted using the ARIS. On the basis of the experimental results, we confirmed that the proposed method effectively obtained the target’s position in 3D space. A moving target simulation was also conducted, and the results showed that this method was effective for moving targets. Finally, a field experiment was performed to obtain the vertical distribution and track the 3D trajectories of fish.

## 1. Introduction

Sonar is a critical tool for underwater obstacle avoidance, bathymetry, acoustic imaging, search, and navigation. Acoustic lens technology provides a relatively compact sensor that can transmit and then receive multiple conical or rectangular beams without using beam-forming electronics [1]. In 2002, a dual-frequency identification sonar (DIDSON) was introduced to the commercial market by Sound Metrics Corp., setting a new standard for excellence in underwater vision in black and turbid waters by obtaining near-video-quality dynamic images for the identification of underwater objects [2]. DIDSON bridged the gap between the existing underwater observation sonars and the optical systems [3]. The Adaptive Resolution Imaging Sonar (ARIS), the next generation of DIDSON, is a useful tool to detect targets within its range with much higher resolution and clarity [4].

**Figure 1:**
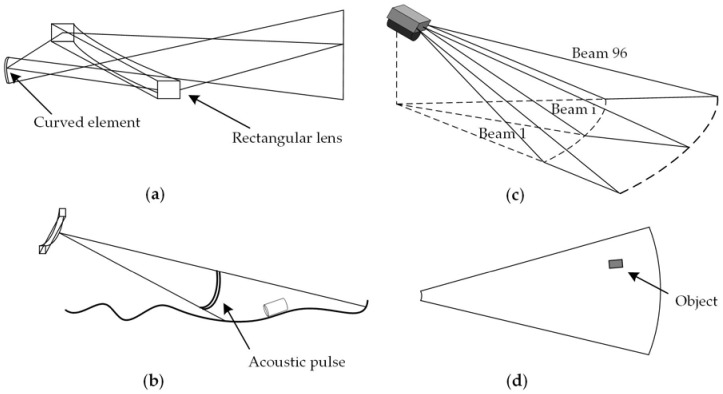
Diagrams of the sonar imaging. (**a**) A line-focus lens system is composed of a rectangular lens and a curved element; (**b**) one beam ensonifies a stripe along the bottom; (**c**) a total of 96 beams are working together; (**d**) an acoustic image acquired from the adaptive resolution imaging sonar (ARIS).

The ARIS is composed of 96 transducer elements forming a linear array. Each element both transmits and receives acoustic beams so that the two-way pattern has a 3 dB beam width of approximately 0.3° [5,6]. Figure 1 displays a sonar imaging diagram. Figure 1a shows one element and one lens, forming together a “line-focused” beam, and Figure 1b shows one beam ensonifying a stripe along the bottom. The element emits an acoustic pulse and receives its echo when it sweeps along the stripe. The echo amplitude is determined by the intensity of the reflected signal. Figure 1c shows how the echoes from all 96 beams map the reflectance of the ensonified sector-shaped area and are used to form an acoustic image, as shown in Figure 1d. To reduce the crosstalk effect when imaging, the 96 transducer elements do not transmit and receive signals simultaneously, but in a specific order. If all the elements are numbered as 1, 2, …, 96 from left to right, they can be divided into 12 groups. The elements in each group transmit beams one by one in a specific order. All groups work in sequence from left to right. Each frame is a composite of an array of the elements working in succession, thereby creating an overall ensemble of partial frames to construct the single frame. However, the difference in time-delays among different ensembles of sound beams may have an impact on the frame construction, especially when the sonar travels at a high speed producing saw-tooth patterns in sonar images [6].

The line-focused beams can produce real-time high-resolution underwater image sequences with a high refresh rate. Moreover, compared with optical devices, the acoustic beams are not affected by turbid or dark waters, ensuring that the scene details and information are properly acquired. However, the ARIS only collects backscattered energy according to the range and azimuth to produce bi-dimensional images. If two objects are in the same range in the same beam with different elevations, the ARIS cannot differentiate them, which prevents obtaining the objects’ positions in 3D space. Negahdaripour et al. proposed methods for system calibration and 3D scene reconstruction using maximum likelihood estimation from noisy image measurements [7]. Brahim et al. used two images of the same scene acquired by a DIDSON from different points of view to reconstruct 3D scenes underwater via evolutionary algorithms [8,9]. Huang and Kaess presented an approach for recovering 3D scene structures from multiple 2D sonar images [10]. With the acoustic images acquired from DIDSON, geometry transformation using different methods helps to effectively reconstruct 3D scenes from a pairwise or multiple viewpoints. A side-scan sonar has also been used for 3D reconstruction. For example, Saucan et al. proposed a novel model-based approach for 3D underwater scene reconstruction using side-scan sonar arrays [11]. Wang et al. used an intensity map acquired by a side-scan sonar to reconstruct the 3D aspects of underwater objects by merging the intensity image and depth image [12]. Most existing studies focused on 3D underwater scene reconstruction, i.e., stationary objects or scenes, with different sonar devices, not on positioning or tracking targets. The studies focusing on tracking underwater targets indicate that imaging sonar is an important tool [13,14]. Handegard et al. used high-resolution sonar (DIDSON) imaging to track the motion and interactions among predatory fish and their schooling prey in a natural environment using 2D images [15]. In this paper, we present a new approach to obtain the target’s 3D coordinates using pairwise images combined with a data association algorithm and we track multiple targets in 3D space using the proposed approach.

The remainder of this paper is organized as follows. The target extraction, data association, and calculation of 3D coordinates are described in Section 2. In Section 3, results from an indoor tank experiment are presented. In Section 4, a simulation of the moving target is outlined. Finally, the results from a field experiment for tracking multiple objects are presented in Section 5, followed by a conclusion in Section 6.

## 2. Materials and Methods

In this section, a method to track multiple targets in 3D space is introduced. First, the signal strength model method is proposed to extract targets from sonar images. An adaptive threshold approach is tested for simultaneous targets detection. After extracting the targets, data association is performed using Multiple Model Joint Probabilistic Data Association (IMMJPDA) algorithm to track same targets in different frames. Then, the missing elevation information for the observed objects within sonar detection range is determined through computing the geometrical transformation between the paired planar images and the cubical space, so that the object’s 3D coordinates are obtained. Finally, the 3D tracks of multiple targets are obtained.

### 2.1. Target Extraction from Sonar Images

Target extraction from a two-dimensional (2D) sonar image is a prerequisite to determine the position of a target in the field of view of the ARIS. Some of the most widely used methods and algorithms for object detection and recognition from images include Haar cascades [16], histograms of oriented gradients [17], and artificial neural networks [18]. Because of low quality, incomplete target visualization, and image distortions caused by acoustic lens imperfections, these methods commonly used in video imagery have limited application for sonar-based target detection. In this study, targets were detected using a newly proposed signal strength model. In the images acquired from the ARIS, the effective target region only contributed minimally, whereas the rest regions were treated as background [19]. First of all, the signal strength model for each pixel was set as:

(1) $$I=\bar{I}+\sigma \sin\left(\omega t\right)+k\zeta$$

where I is the intensity value for this pixel, $\bar{I}$ is the average intensity value of this image, σ is the intensity amplitude of the background, ζ is the noise level, k is the coefficient of noise level that usually equals 1, and ω and t are the intensity vibrational angular frequency and time, respectively. When I is satisfied with the formula below, it can be treated as background:

(2) $$\bar{I}-\sigma -\zeta \leq I\leq \bar{I}+\sigma +\zeta$$

Because the intensity of a target is larger than that of the background, the target can be selected using the formula below:

(3) $$I>\bar{I}+\sigma +\zeta$$

**Figure 2:**
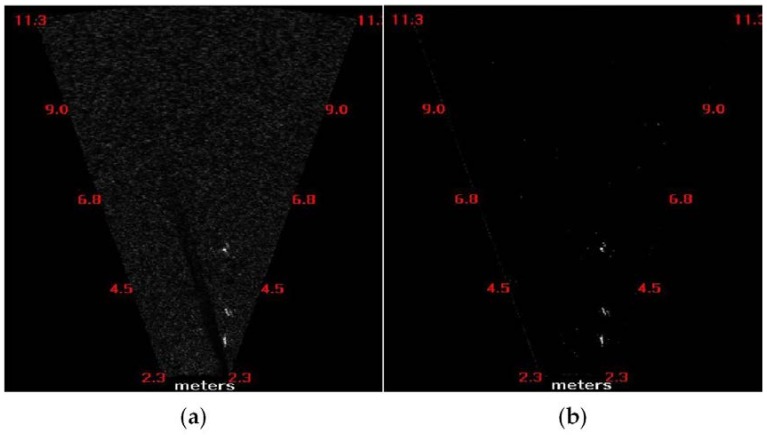
Target extraction with the proposed method. (**a**) Raw image and (**b**) image after background elimination. The bright regions in (**b**) are the targets.

$\bar{I}$ and σ were updated in every image sequence according to Equations (3) and (4).

(4) $${\bar{I}}^{\prime }=\frac{\left(n-1\right)}{n}\bar{I}+\frac{1}{n}I$$

(5) $$\sigma^{\prime }=\frac{\left(n-1\right)}{n}\sigma +\frac{1}{n}\sqrt{2{\left(I-\bar{I}\right)}^{2}}$$

where ${\bar{I}}^{\prime }$ is the new intensity value, $\sigma^{\prime }$ is the new intensity amplitude of the background, and n is the iterative coefficient. Figure 2 depicts the target extraction using the proposed method, in which $\bar{I}$ equals 15, ξ is 30, and n equals 5.

For a complex background, we tested an adaptive threshold approach for target extraction. Because the higher pixel values in sonar image are the potential targets that need to be detected, a threshold T was set to distinguish the targets from the background. When the pixel value v(x,y)<T, it will be labeled as background; otherwise, the pixel is the target. We used the following method to select the threshold T [20].

Assuming that a pixel value v(x,y) in the *k*th frame is subjected to Gaussian distribution with the mean μ and the variance $\sigma^{2}$ in continuous frames: $v\left(x,y\right)∼N\left(\mu ,\sigma^{2}\right)$. According to the thrice standard error principle, the probability that v(x,y) lies outside the range of [−3σ,3σ] is less than 0.3%; hence, T=μ + β⋅3σ, in which β is the coefficient of the threshold. Additionally, by averaging the mean and variance of several consecutive frames as the final mean μ and variance $\sigma^{2}$, the Gaussian distribution function is determined, so that T is obtained. 

In optical image processing, edge detection is frequently used for target extraction. However, a sonar image is generated from a 2D array data acquired by 96 transducer elements through coordinate transformation and data interpolation. During the frame construction, data interpolation definitely reduces the sharpness of the image. Additionally, the boundary of the sonar image is not clear because of speckle noise [21]. Hence, general edge detection is not suitable for target extraction during sonar image processing, but some special or high-quality edge detection algorithms are useful for target detection [22]. 

Using the target extraction algorithms proposed in this section, the bright regions are detected. Conveniently, to track targets in 3D space, the underwater target is regarded as a point target to avoid the influence of target size change. The equation below provides a method to label a target with the coordinates $\left(x_{t},y_{t}\right)$:

(6) $$m_{a,b}=\sum_{x}\sum_{y}x^{a}y^{b}v\left(x,y\right)$$

where a = 0 or 1, and b = 0 or 1. Hence, the target coordinates can be obtained: $\left(m_{1,0}/m_{0,0},m_{0,1}/m_{0,0}\right)$.

**Figure 3:**
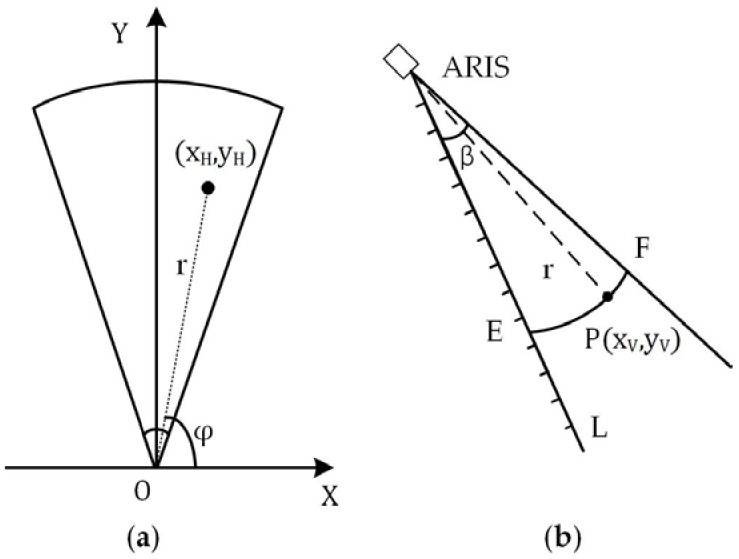
(**a**) Horizontal field of view of the sonar; (**b**) vertical field of view of the sonar. β is the angle of the vertical field of view, which is approximately 14°.

Figure 3a shows the coordinates of the target $\left(x_{H},y_{H}\right)$ in the horizontal field of view of the sonar. With the range r and azimuth φ, the coordinates can be obtained: $x_{H}=r\cdot \cos\varphi$, $y_{H}=r\cdot \sin\varphi$.

### 2.2. Data Association

Because of the randomness of underwater target movement, especially when the targets are underwater fish, Interactive Multiple Models (IMM) combined with Joint Probabilistic Data Association (JPDA) filtering is proposed to correlate the same target in different images [23], so that a target appearing in different frames can be tracked, and these appearances can be connected as one target. The data association proposed is used not only to examine the overlapping portion of two consecutive images but also to calculate the 3D position of the targets.

First, the target motion models are established, including the Brownian motion model, constant velocity (CV) model, and constant acceleration (CA) model. The jump rules among these three models obey the Markov chain for which the transfer probability is known [24]. 

Assuming that, in the *k*th frame image, N targets ${\left\{T_{i}\right\}}_{i=1}^{N}$ are extracted, and each target corresponds to a motion model $M_{j}$(j=1,⋯,n,n=3), the motion equation and measurement equation of the target r are described as follows:

(7) $$\left\{ \begin{array}{l} x_{k}=F_{k-1}^{j}x_{k-1}+G_{k-1}^{j}W_{k-1}^{j}\\ z_{k}=H_{k}^{j}x_{k}+G_{k-1}^{j}V_{k}^{j} \end{array}\right.$$

where $x_{k}$ is the state of the target r at time k, $z_{k}$ is the observation vector, $F_{k-1}^{j}$ is the state of the transform matrix at time k−1, $H_{k}^{j}$ is the measurement matrix, $G_{k-1}^{j}$ is the input matrix, and $W_{k-1}^{j}$ and $V_{k}^{j}$ are uncorrelated Gaussian white noises with zero mean corresponding to the covariance $Q_{k-1}^{j}$ and $R_{k}^{j}$, respectively.

The state of the target $x_{k}$ includes position, velocity, and acceleration in each of the two Cartesian coordinates (x and y). The state of the transform matrix F can be defined as [25]:

(8) $$F=\left[\begin{array}{cc} F_{b} & 0\\ 0 & F_{b} \end{array}\right]$$

Hence, the Brownian motion model is given by:

(9) $$F_{b}^{1}=\left[\begin{array}{ccc} 1 & 0 & 0\\ 0 & 0 & 0\\ 0 & 0 & 0 \end{array}\right]$$

The CV model with zero mean perturbation in acceleration is:

(10) $$F_{b}^{2}=\left[\begin{array}{ccc} 1 & T & 0\\ 0 & 1 & 0\\ 0 & 0 & 0 \end{array}\right]$$

The CA model is:

(11) $$F_{b}^{3}=\left[\begin{array}{ccc} 1 & T & T^{2}/2\\ 0 & 1 & T\\ 0 & 0 & 1 \end{array}\right]$$

Target tracking is realized as follows:

(1) State initialization:
  (12) $${\hat{x}}_{k-1|k-1}^{0j}=E\left\{x_{k-1}|M_{k}^{j},Y_{k-1}\right\}=\sum_{i=1}^{n}{\hat{x}}_{k-1|k-1}^{i}\mu^{i|j}$$
  (13) $$p_{k-1|k-1}^{0j}=\sum_{i=1}^{n}p_{k-1|k-1}^{j}+\left\{\left[{\hat{x}}_{k-1|k-1}^{j}-{\hat{x}}_{k-1|k-1}^{0j}\right]{\left[{\hat{x}}_{k-1|k-1}^{j}-{\hat{x}}_{k-1|k-1}^{0j}\right]}^{\prime }\right\}\mu^{i|j}$$
  where E{⋅} is the mathematical expectation, $Y_{k-1}$ is the cumulative set of measurements up to time k−1, $\mu^{i|j}$ is the mixing probability when the motion mode changes from $M_{i}$ to $M_{j}$, ${\hat{x}}_{k-1|k-1}^{0j}$ is the mixed estimate, and $p_{k-1|k-1}^{0j}$ is the covariance of the mixed estimate.
(2) State prediction:
  (14) $${\hat{x}}_{k|k-1}^{j}=F_{k-1}^{j}x_{k-1|k-1}^{0j}$$
  (15) $$P_{k|k-1}^{j}=F_{k-1}^{j}P_{k-1|k-1}^{0j}{\left(F_{k-1}^{j}\right)}^{T}+G_{k-1}^{j}Q_{k-1}^{j}{\left(G_{k-1}^{j}\right)}^{T}$$
  where $P_{k|k-1}^{j}$ is the state prediction error covariance. The residual error corresponding to measurement i is:
  (16) $${\tilde{z}}_{k}^{j,\left(i\right)}=z_{k}^{\left(i\right)}-H_{k}^{j}{\hat{x}}_{k|k-1}^{j}$$
  The covariance of the residual is given by:
  (17) $$S_{k}^{j}=H_{k}^{j}P_{k|k-1}^{j}{\left(H_{k}^{j}\right)}^{T}+R_{k}^{j}$$
(3) Association probability update:
  (18) $$\beta_{i}^{r,j,J}=\sum_{\theta }p\left\{\theta |Y_{k}\right\}{\hat{\omega }}_{ir}\left(\theta \right)$$
  where $\beta_{i}^{r,j,J}$ represents the posterior probability, given by the measurement i connected with the target r using the motion model set J, and θ is the joint events set. J is a set corresponding to the targets $r_{⊳}\left(r_{⊳}\neq r\right)$ motion models, except r. $\beta_{i}^{r,j}$ is given by:
  (19) $$\beta_{i}^{r,j}=\sum_{J}\mu_{k-1}^{J}\beta_{i}^{r,j,J},r=0,1,\cdots ,N$$
  (20) $$\mu_{k-1}^{J}=\prod_{r_{⊳}=1}^{N}\mu_{k-1}^{j}\left(r_{⊳}\right),r_{⊳}\neq r$$
  where $\mu_{k-1}^{j}\left(r_{⊳}\right)$ is the probability corresponding to the target $r_{⊳}$ with model $M_{j}$ at time k−1.
(4) State update with different models. Kalman gain is given by
  (21) $$W_{k}^{j}=P_{k|k-1}^{j}{\left(H_{k}^{j}\right)}^{T}{\left(S_{k}^{j}\right)}^{-1}$$
  The state vector ${\hat{x}}_{k}^{j}$ is updated with different motion models:
  (22) $${\hat{x}}_{k|k}^{j}={\hat{x}}_{k|k-1}^{j}+W_{k}^{j}{\tilde{y}}_{k}^{r,j}$$
  where
  (23) $${\tilde{y}}_{k}^{r,j}=\sum_{i=1}^{\bar{m}}\beta_{i}^{r,j}{\tilde{z}}_{k}^{j,\left(i\right)}$$
  The state prediction error covariance is updated:
  (24) $$P_{k|k}^{j}=P_{k|k-1}^{j}-\left(\sum_{i=1}^{\bar{m}}\beta_{i}^{r,j}\right)W_{k}^{j}S_{k}^{j}{\left(W_{k}^{j}\right)}^{T}+W_{k}^{j}\left[\sum_{i=1}^{\bar{m}}\beta_{i}^{r,j}{\tilde{z}}_{k}^{j,\left(i\right)}{\left({\tilde{z}}_{k}^{j,\left(i\right)}\right)}^{T}-{\tilde{y}}_{k}^{r,j}{\left({\tilde{y}}_{k}^{r,j}\right)}^{T}\right]{\left(W_{k}^{j}\right)}^{T}$$
(5) The likelihood function is updated:
  (25) $$\Lambda_{k}^{r,j}=N\left\{z\left(k;{\hat{x}}_{k|k}^{j}\right),\hat{z}\left(k|k-1;{\hat{x}}_{k-1|k-1}^{0j}\right),S\left(k;P_{k-1}^{0j}\right)\right\}$$
  where N{⋅}represents the normal distribution.
(6) The model probability is updated:
  (26) $$\mu_{k}^{j}\left(r\right)=\frac{1}{c}\mu_{k-1}^{j}\left(r_{⊳}\right)\Lambda_{k}^{r,j}$$
  where c is a normalization constant given by:
  (27) $$c=\sum_{j=1}^{r}\mu_{k-1}^{j}\left(r_{⊳}\right)\Lambda_{k}^{r,j}$$
(7) Target state update:
  (28) $${\hat{x}}_{k|k}=\sum_{j=1}^{r}{\hat{x}}_{k|k}^{j}\mu_{k}^{j}$$
  The state prediction error covariance of ${\hat{x}}_{k|k}$ is:
  (29) $$P_{k|k}=\sum_{j=1}^{r}\mu_{k}^{j}\left[P_{k|k}^{j}+\left({\hat{x}}_{k|k}^{j}-{\hat{x}}_{k|k}\right)\cdot {\left({\hat{x}}_{k|k}^{j}-{\hat{x}}_{k|k}\right)}^{T}\right]$$

**Figure 4:**
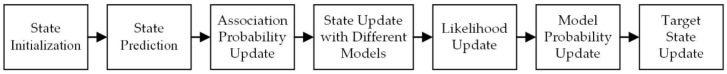
Flow chart of the Interactive Multiple Model–Joint Probabilistic Data Association (IMM–JPDA).

The flow chart of IMM–JPDA filtering is shown in Figure 4. 

**Figure 5:**
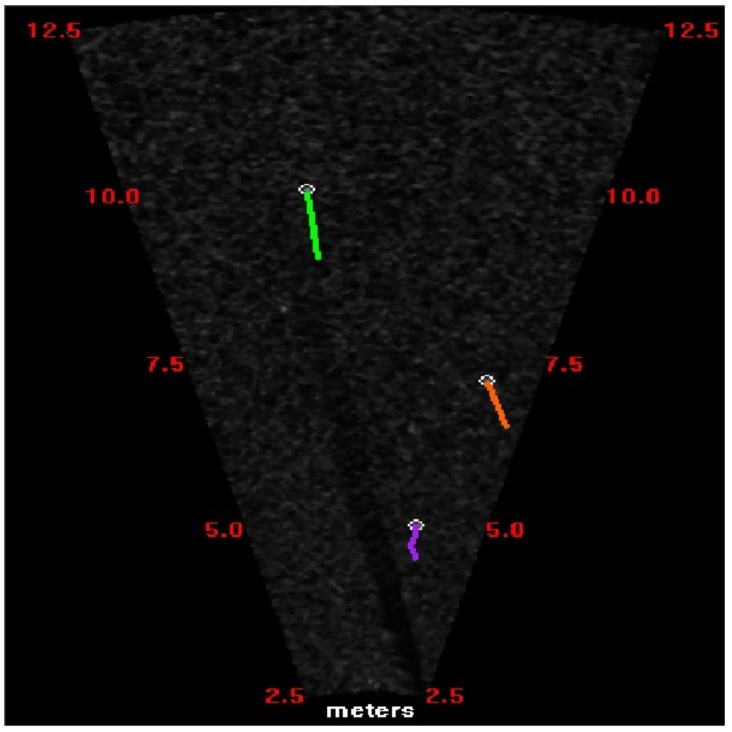
Diagram of tracking trajectories of multiple targets. Three different colored lines represent three different target trajectories.

Using the data association algorithm, the same targets from different image sequences are connected. When the positions of the targets from different frames are preserved, different target trajectories are identified with different colors, as shown in Figure 5. Compared to other data association or tracking algorithms, such as the nearest neighbor (NN) algorithm or Kalman filtering (KF), IMM–JPDA has more advantages. First, the target motion models of IMM–JPDA are more appropriate than in KF, since uniform linear motion is set for KF. Also, the JDPA data correlation calculation is more accurate than with the NN algorithm. 

To verify the accuracy of target extraction and tracking in a complicated background, the test data were manually determined. This test data had a total of 144 frames, from which 5971 candidate targets were detected manually, whereas 5901 were extracted using the signal strength model method with a 1.2% error rate, and 5882 were extracted using the adaptive threshold approach with an error rate of 1.5%. Simultaneously, 497 fish were counted manually, and 468 were counted using the tracking algorithm with 5.8% error. Here, a candidate target was a bright region extracted from the sonar image (Figure 2b); hence, the statistics of candidate target numbers is an accumulation of bright regions from all frames. However, a fish target must satisfy the conditions of live targets, including length, width, and swimming speed. One fish represents one track trajectory from presence to absence in several continuous frames.

### 2.3. Calculation of 3D Coordinates 

Assume a target $P\left(x_{V},y_{V}\right)$ appears in the vertical field of view of the sonar, as shown in Figure 3b. Given the range r$\left(x_{V}^{2}+y_{V}^{2}=r^{2}\right)$ and the azimuth φ of this beam, the ARIS could not exactly obtain the coordinates $\left(x_{V},y_{V}\right)$, because P may appear anywhere in the arc EF, resulting in a failure to obtain the target’s position in 3D space.

Because of the movement of the ARIS, the two target positions extracted from sonar images obtained at different locations are different from each other. With the computation of the geometrical transformation between the acquisition planar images and the cubical space, the 3D coordinates of the target can be obtained from the two different target positions. In the actual survey, the ARIS was always mounted on a vessel with the same transmission direction as the beam direction, or perpendicular to the beam direction. These two cases are discussed below.

#### 2.3.1. Case 1: ARIS Moves along the Beam Transmitting Direction

**Figure 6:**
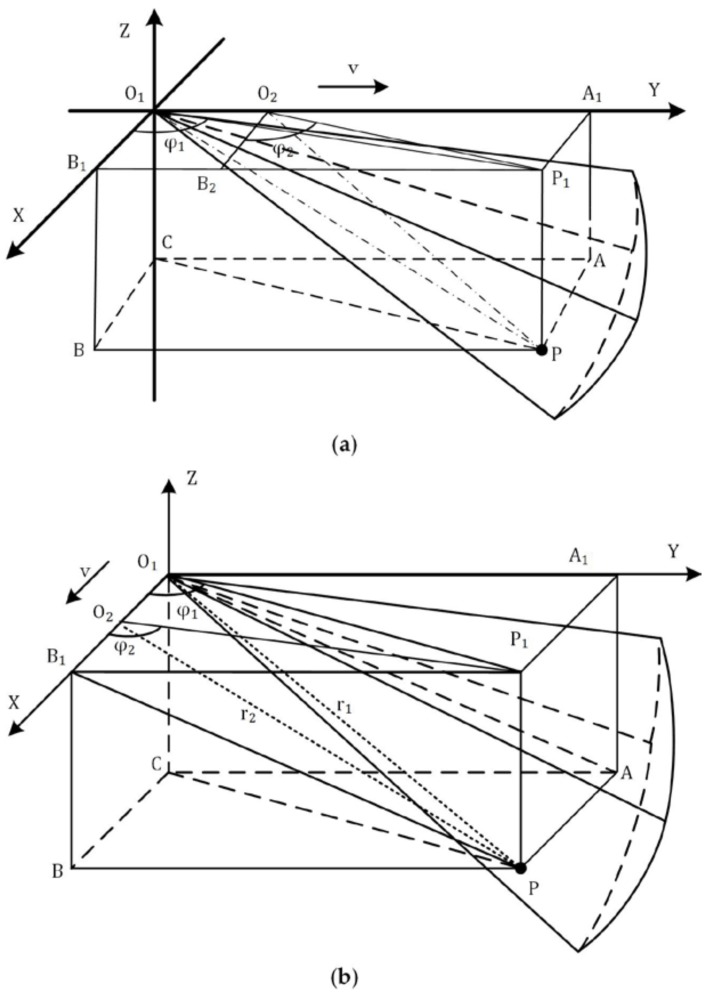
Two detection modes. (**a**) The Adaptive Resolution Imaging Sonar (ARIS) moves along the beam transmitting direction; (**b**) the moving direction of the sonar is perpendicular to the beam transmitting direction.

Suppose that the ARIS moves along the *y*-axis and transmits multi-beams along the *y*-axis with a certain angle below the plane $XO_{1}Y$, as shown in Figure 6a. In this figure, $O_{1}$ is the location where the ARIS stays at time $t_{1}$, $O_{2}$ is the location where the ARIS stays at time $t_{2}$, point P(x,y,z) is the object needing to be positioned. To facilitate the understanding and calculation of the coordinates, the cuboid $APBC-A_{1}P_{1}B_{1}O_{1}$ is established, in which $O_{1}P$ is the body diagonal, A is the projection of P on the plane $YO_{1}Z$, B is the projection of P on plane $XO_{1}Z$, and $P_{1}$ is the projection of P on plane $XO_{1}Y$. In this cuboid, we set $\left|O_{1}P\right|=r_{1}$, $\left|O_{2}P\right|=r_{2}$, $∠B_{1}O_{1}P_{1}=\varphi_{1}$, and $∠B_{2}O_{2}P_{1}=\varphi_{2}$, in which $\left(r_{1},\varphi_{1}\right)$ and $\left(r_{2},\varphi_{2}\right)$ were the target coordinates extracted from site $O_{1}$ and $O_{2}$, respectively. 

$O_{1}P_{1}$ and $O_{2}P_{1}$ are the projections of $O_{1}P$ and $O_{2}P$ on plane $XO_{1}Y$, respectively. $\varphi_{1}$ is the azimuth of the target at site $O_{1}$, and $\varphi_{2}$ is the azimuth of the target at site $O_{1}$. Hence, we can obtain the coordinates of $P_{1}$ by calculating the intersection of $O_{1}P_{1}$ and $O_{2}P_{1}$, as shown in the equations below:

(30) $$\left\{ \begin{array}{l} L_{O_{1}P_{1}}:y=\tan\varphi_{1}\cdot x\\ L_{O_{2}P_{1}}:y=\tan\varphi_{2}\cdot x+\delta \end{array}\right.$$

where δ is the distance between $O_{1}$ and $O_{2}$, δ=v⋅Δt, in which Δt is the time gap, and v is the moving speed of the ARIS. We can obtain the solution:

(31) $$\left\{ \begin{array}{l} x=\delta /\left(\tan\varphi_{1}-\tan\varphi_{2}\right)\\ y=\delta \cdot \tan\varphi_{1}/\left(\tan\varphi_{1}-\tan\varphi_{2}\right)\\ z=-\sqrt{r_{1}^{2}-x^{2}-y^{2}} \end{array}\right.$$

#### 2.3.2. Case 2: ARIS Moves Perpendicular to the Beam Transmitting Direction

Suppose that the ARIS transmits multi-beams along the *y*-axis with a certain angle below the plane $XO_{1}Y$ and moves along the *x*-axis at a constant speed v, as shown in Figure 6b. $O_{1}$ and $O_{2}$ are the locations where the ARIS stays at times $t_{1}$ and $t_{2}$, respectively. Point P(x,y,z) is the object needing to be positioned. As in Figure 6a, the cuboid $APBC-A_{1}P_{1}B_{1}O_{1}$ is established, in which $\left|O_{1}P\right|=r_{1}$, $\left|O_{2}P\right|=r_{2}$, $∠B_{1}O_{1}P_{1}=\varphi_{1}$, and $∠B_{1}O_{2}P_{1}=\varphi_{2}$. We can obtain the coordinates of $P_{1}$ from the equations below:

(32) $$\left\{ \begin{array}{l} L_{O_{1}P_{1}}:y=\tan\varphi_{1}\cdot x\\ L_{O_{2}P_{1}}:y=\tan\varphi_{2}\cdot \left(x-\delta \right) \end{array}\right.$$

Thus, the coordinates of point P(x,y,z) are:

(33) $$\left\{ \begin{array}{l} x=\delta \cdot \tan\varphi_{2}/\left(\tan\varphi_{2}-\tan\varphi_{1}\right)\\ y=\delta \cdot \tan\varphi_{1}\cdot \tan\varphi_{2}/\left(\tan\varphi_{2}-\tan\varphi_{1}\right)\\ z=-\sqrt{r_{1}^{2}-x^{2}-y^{2}} \end{array}\right.$$

Combining the 3D coordinate calculation with the data association algorithm, the target can be tracked in 3D space. Firstly, the coordinates $\left(r_{1},\alpha_{1}\right),\left(r_{2},\alpha_{2}\right),\cdots ,\left(r_{n},\alpha_{n}\right)$ are extracted from n frame images. Secondly, the target coordinates $\left(x_{k},y_{k},z_{z}\right)$ at time k can be obtained from $\left(r_{k},\alpha_{k}\right)$ and $\left(r_{k+1},\alpha_{k+1}\right)$, in which k=1,2,⋯,n−1. Finally, the target trajectory can be acquired by connecting the n−1 points.

## 3. Indoor Water Tank Experiment

**Figure 7:**
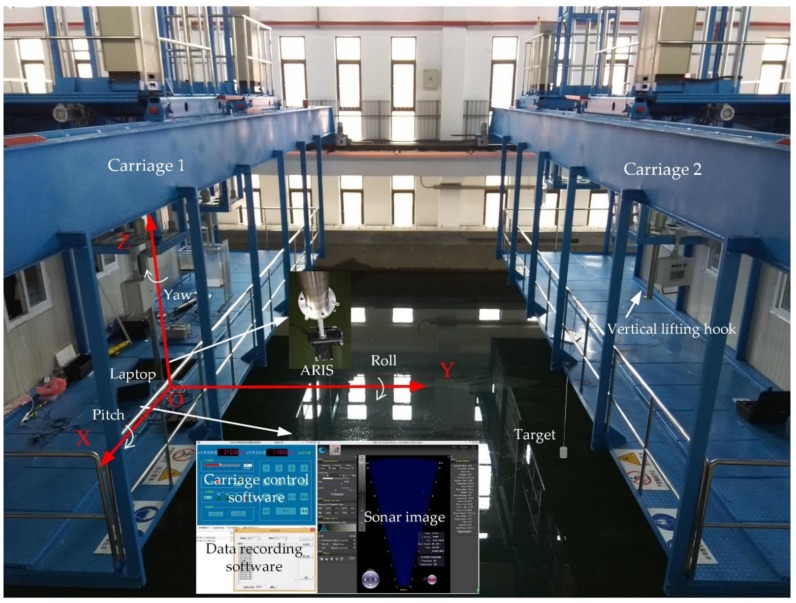
Photo of the indoor experiment.

An experiment was performed to evaluate the accuracy of the proposed approach for obtaining the target’s 3D position using an imaging sonar. The experiment was conducted in a pool with a length of 50 m, width of 15 m, and depth of 10 m. The water depth in this experiment was approximately 9 m. Two carriages were present above the pool, and each carriage had a platform with two vertical lifting hooks. The ARIS was fixed on one of the vertical lifting hooks on carriage 1, accessed by a laptop, and was submerged under water just until completely covered, with a pitch angle of 10° downward (Figure 7). Carriage 1 moved in the same direction as the sonar beam transmitting direction, controlled by carriage control software running in the laptop, and the state information of this carriage, including the location and time, was also recorded by another program running in this laptop. The target was hung under water on carriage 2. The target was a metal cylinder with a bottom diameter of 54 mm and a height of 107 mm.

In Figure 7, the displayed space coordinate system was established with the ARIS located at the origin of the coordinates. Carriage 1 moved along the y-axis at a constant velocity of no more than 1.5 m/min, whereas the target hung on carriage 2 was static and underwater. When the test started, sonar data and carriage information were recorded simultaneously. After data collection, the target’s 3D coordinates and the errors compared to the measured values were calculated. Four datasets were recorded and they are listed in Table 1. Because of the limitation of the detection range under the high frequency working mode of the ARIS, the coordinates of the target (x,y,z) were in the range of |x|<3.6 m, |y|<15 m, and |z|<6 m. In this test, we set the target coordinates $\left(1.30,y_{meas},-3.23\right)$, in which $y_{meas}$ was determined by the location of carriage 1.

**Figure 8:**
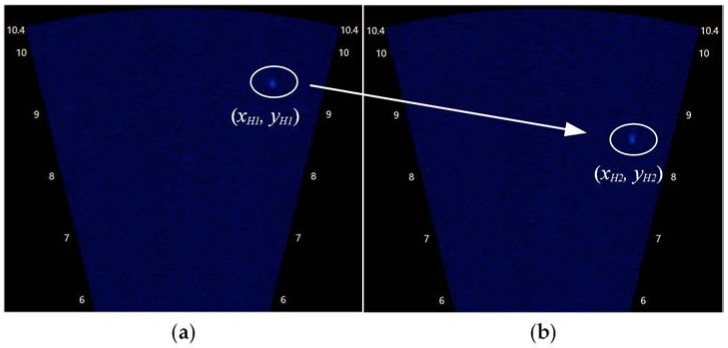
The sonar images corresponding to the data of condition No. 2 in Table 1. (**a**) Image acquired at time 1; (**b**) image acquired at time 2.

As shown in Table 1, strong agreement was demonstrated between the calculated and the measured coordinates. Figure 8 shows the sonar images that correspond to the data of condition No. 2 in Table 1.

Many factors can lead to errors. The carriage moving on the rail caused a small vibration, causing the sonar fixed on the vertical lifting hook to shake as well. The length of this hook was more than three meters; therefore, the target’s position extracted from the sonar images would be inaccurate. Additionally, the roll angle of the sonar was not exactly equal to zero, and the yaw angle of the sonar was not exactly toward the *y*-axis (Figure 7). A small deviation in the roll or yaw angle may cause a considerable positional error, which was inevitable. In addition, the deviation between the measured coordinates and the real target’s position was non-negligible. With the ARIS moving, cross-talk was detrimental to target extraction, especially when the target was near to the ARIS, creating a source of error.

## 4. Simulation on Moving Target

Underwater targets, like fish, are not static, even if the interval between two frames is very short, which leads to positioning error. Hence, the analysis of the calculation error using the proposed method is necessary when the target moves at different speeds and in different directions. Assuming that the sonar is displayed as in case 2 (Figure 6b), the target is located at point $P_{1}\left(x_{1},y_{1},z_{1}\right)$ at time $t_{1}$, and at point $P_{2}\left(x_{2},y_{2},z_{2}\right)$ at time $t_{2}$. Taking the intermediate point as the real position during the time $t_{1}\sim t_{2}$, we can obtain:

(34) $$P_{real}\left(x_{real},y_{real},z_{real}\right)=\left(\frac{x_{1}+x_{2}}{2},\frac{y_{1}+y_{2}}{2},\frac{z_{1}+z_{2}}{2}\right)$$

Supposing that the coordinates obtained from calculation are $P_{cal}\left(x_{cal},y_{cal},z_{cal}\right)$, the positional error is defined as below:

(35) $$d_{err}=\left|P_{cal}-P_{real}\right|/\left(\frac{r_{1}+r_{2}}{2}\right)\times 100\%$$

where $\left|P_{cal}-P_{real}\right|$ represents the distance between the real position and the calculated position.

**Figure 9:**
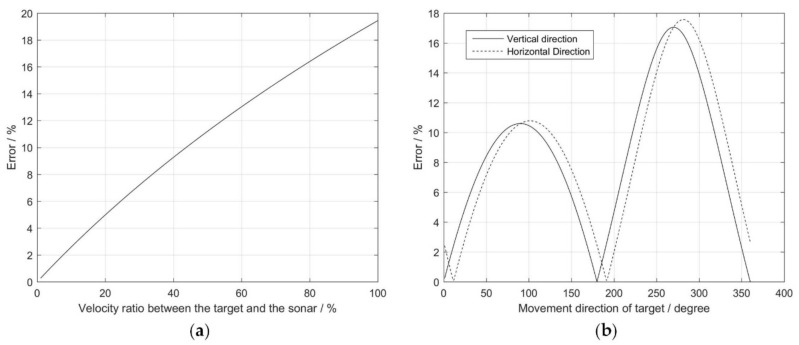
The influence of different factors on the positioning error: (**a**) Velocity ratio between the target and the sonar; and (**b**) target’s moving direction, including vertical direction and horizontal direction.

Given $P_{1}\left(x_{1},y_{1},z_{1}\right)=P_{1}\left(10,2,-4\right)$, δ=0.1, and $\left\{ \begin{array}{l} x_{2}=x_{1}+d\cdot \sin\theta \cdot \cos\alpha \\ y_{2}=y_{1}+d\cdot \sin\theta \cdot \sin\alpha \\ z_{2}=z_{1}+d\cdot \cos\theta \end{array}\right.$, where the spherical coordinates (d,θ,α) are used to describe the target’s movement $\vec{P_{1}P_{2}}$. Figure 9 shows the influences of different factors on the positioning error. When the target moves in a certain direction $\theta =160^{\circ }$ and $\alpha =15^{\circ }$, the error increases as the velocity ratio between the target and sonar increases (Figure 9a). When the target moves with a certain velocity, d/δ=0.01, the error is periodic, regardless of whether the vertical direction or the horizontal direction of the target change (Figure 9b). From Figure 9, the positional error arises as a consequence of the greater velocity ratio between the target and the sonar. The influence of the target’s velocity and direction on the positioning error is less than 20%. 

In addition to the target movement, the target position corresponding to the sonar position also impacts the positional error. To determine the relationship between the positional error and grazing angle, a simulation was conducted.

**Figure 10:**
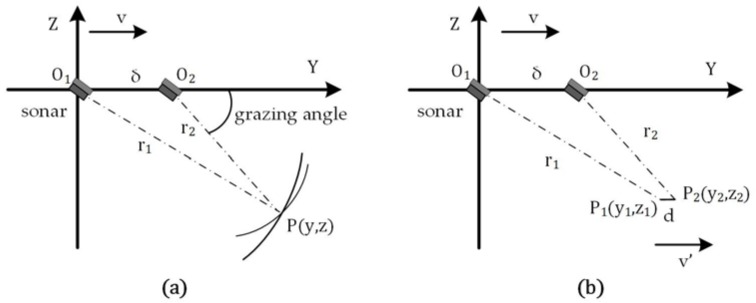
Diagram of the target and sonar in different states. (**a**) The target is stable while the sonar moves; (**b**) the target and the sonar move simultaneously.

**Figure 11:**
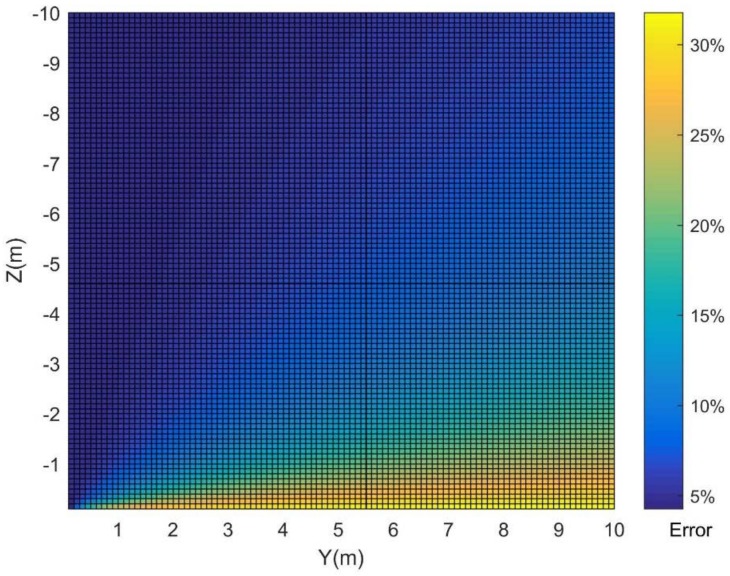
The target position corresponding to the sonar position impacts the positioning error, which is the error distribution when the target moves at a specified speed.

Assuming that the sonar is displayed as in case 2 shown in Figure 10, the target moves in the plane *YOZ* and is located at point $P_{1}\left(x_{1},y_{1},z_{1}\right)$ at time $t_{1}$, and at point $P_{2}\left(x_{2},y_{2},z_{2}\right)$ at time $t_{2}$. Given the spherical coordinates (d,θ,α) between $P_{1}$ and $P_{2}$ as (0.01,π/2,π/2) and δ=0.1, the measurement error of the distance is 5%, and the positional error $d_{err}$ is defined by Equation (35). When the target position corresponds to the sonar changes in the plane *YOZ*, the positional error is obtained as shown in Figure 11. From this figure, the error increases with the increase in the *Y* value or the *Z* value but stays almost the same regardless of the *Z* value when the *Y* value is less than 1. In other words, the error decreases as the grazing angle increases.

## 5. Field Experiment

A field experiment to track fish in 3D space was performed in Dishui Lake (121°56′ E, 30°54′ N), on the basis of the proposed method. Dishui Lake is the largest artificial lake in Shanghai, China. The lake is round in shape, approximately 2.6 km in diameter, with an area of 5.56 km^2^. Water in this lake comes from the Huangpu River via the Dazhi River through surrounding river networks, accepts surface runoff, and passes through a sluice into the East China Sea. The lake is important for flood control, drainage, and water replacement, and is critical to Shanghai's eco-city construction. It maintains several freshwater species, including silver carp and spotted silver carp. The body lengths of most fish are more than 20 cm, and the typical size is 40 cm.

**Figure 12:**
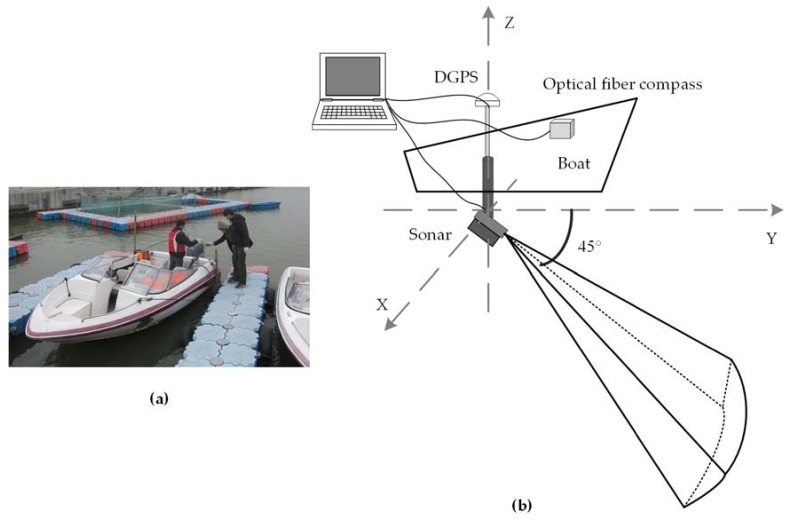
Sonar installation. (**a**) Measurement vessel and equipment installation in the field experiment; (**b**) diagram of the sonar installation.

In this experiment, the sonar was mounted 0.5 m underwater on the side of a boat, with a pitch angle of 45° downward. The detection direction was along the *y*-axis, in the same direction as the boat movement (Figure 12). The velocity of the boat was 2 knots. The GPS module (DGPS) and the attitude sensor (optical fiber compass) were accessed by a laptop. The optical fiber compass included an attitude sensor to obtain the attitude of the sonar in real time and to improve the sonar image. If the attitude of the sonar exceeded the reasonable scope, the sonar data were abandoned.

After data collection, the fish were mainly detected with the signal strength model method. When the background was complex, the adaptive threshold approach was used to extract the fish targets. To distinguish the fish target from noise, a size threshold was set according to the live fish in Dishui Lake: if the bright region of a candidate target was no less than 20 cm in length and 4 cm in width and no more than 80 cm in length and 20 cm in width, it was regarded as a fish target, otherwise it was regarded as an other target. 

IMM–JPDA filtering was applied to associate the fish targets extracted from consecutive frames, once the targets represented one fish. Hence, the depth of the target was obtained using the algorithm proposed in this paper, so that the 3D coordinates of each target were also acquired. Each target appeared in several continuous frames, from presence to absence. The target trajectory sequentially connects the positions of one target from different frames with line segments. 

**Figure 13:**
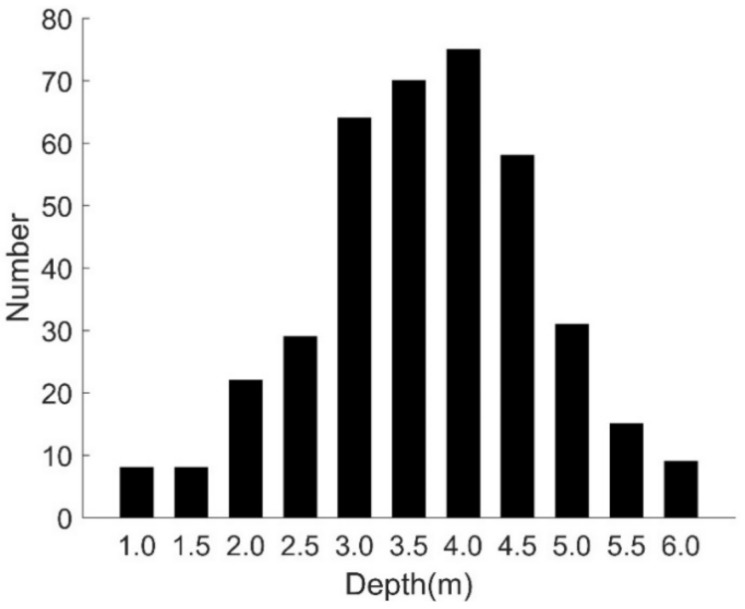
Fish distribution at different water depths.

We recorded a dataset covering a period of 10 min and ran statistics on fish vertical distribution according to depth, as shown in Figure 13. A total of 391 fish were counted in this dataset, and most of the fish swam at depths of three to five meters. 

**Figure 14:**
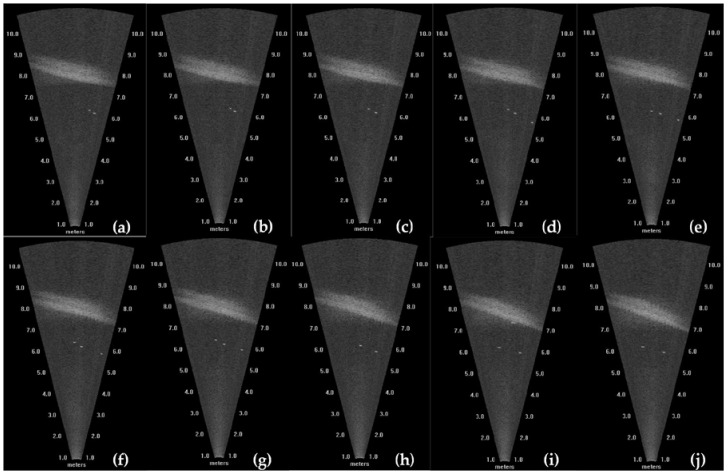
Charts of 10 consecutive frames. (**a**–**j**) represent these 10 consecutive frames.

**Figure 15:**
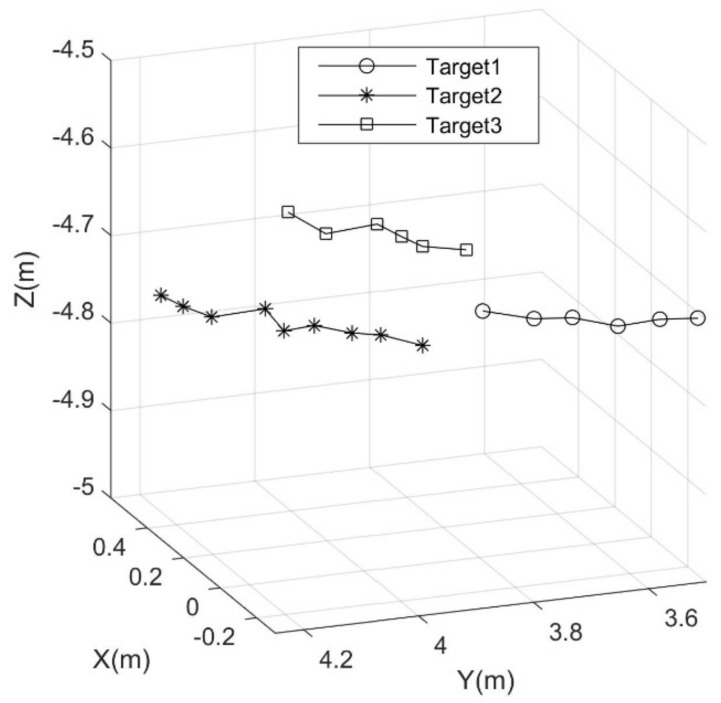
Target trajectories in 3D space corresponding to the 10 consecutive frames in Figure 14.

Ten consecutive frame images were selected to calculate the targets’ 3D coordinates, as shown in Figure 14. With the method proposed in this study, the target trajectories were obtained, as shown in Figure 15. This figure shows three tracks in 3D space, and different types of lines represent different tracks.

To evaluate the accuracy of the 3D tracking of live targets, the velocity and direction of the moving targets, together with the grazing angle of the sonar, were considered. On the basis of the data in Figure 13, the mean velocity of the targets was calculated and was approximately 0.5 m/s, and the positional error caused by target movement was about 10% compared with the velocity of the vessel. As for the moving direction, as shown in Figure 15, the error was less than 5%. As for the grazing angle of the sonar, the error was approximately 10% (Figure 11). Hence, the standard deviation of all errors was: $\sigma =\sqrt{\left(0.1^{2}+0.05^{2}+0.1^{2}\right)/3}\approx 8.7\%$. However, the vibration of the sonar was not considered, so the error in the field experiment was probably larger.

There are many possible causes for the error on the 3D track. (1) For target detection and tracking, when the targets are dense or the signal-to-noise ratio (SNR) of the targets is low, multiple objects overlap, leading to a fatal error in target detection and tracking. When the fish are milling or close to a rugged bottom, the tracker may break up long tracks. When the fish are traveling very close together in the form of a large group along a route, they may not be perceived as separate targets by the tracker. The velocity of the vessel may also have been too high for some routes during the field experiment, and the images collected from a moving sonar are commonly susceptible to smearing because of transient effects and noise, which cause interference in the process of target identification and tracking. Conversely, the high velocity of the fish will produce the Doppler Effect, which leads to error in target detection and positioning; (2) For 3D coordinate calculation, the positioning error is influenced by the velocity and direction of the moving target and the grazing angle of the sonar. Compared with the experiment in the indoor tank, the positional error in the field experiment may have been greater as a result of the rocking of the vessel, causing the sonar mounted on the vessel to sway simultaneously. The sonar may also sense the vibration caused by the vessel’s engine. 

To obtain better experiment results, the following operations can be conducted to reduce the error. First, the pitch angle of the sonar should be as large as possible, the nearer is the pitch angle to 90°, the less error caused by the grazing angle will be produced. Second, the sonar should be installed far away from the engine to reduce the influence of the bubbles caused by the propeller. Third, a calm weather is preferable for a field experiment, so that the vessel can move as smoothly as possible to reduce the fluctuation in the sonar images.

## 6. Conclusions

In this study, a method to track underwater targets in 3D space using an imaging sonar was proposed. An indoor experiment was performed to verify the feasibility and accuracy of this method. The results showed that this method was capable of positioning a target in space. A data association algorithm was designed to track underwater targets in planar images. Combining the positioning method with the data association algorithm, the spatial locations of targets were obtained. Finally, a field experiment was conducted to obtain the 3D trajectories of multiple targets. In conclusion, the proposed approach provides a new method for underwater tracking in 3D space in turbid or dark water, which is helpful for the evaluation of fishery resources.

## Figures and Tables

**Table 1:** Data acquired from this experiment. $\left(x_{H1},y_{H1}\right)$ and $\left(x_{H2},y_{H2}\right)$ are the target’s coordinates extracted from the two sonar images acquired at different positions; $d_{1}$ and $d_{2}$ are the locations of carriage 1; (x,y,z) are the 3D coordinates of the target calculated with the proposed method, and $\left(x_{err},y_{err},z_{err}\right)$ are the errors of (x,y,z) compared with $\left(1.30,y_{meas},-3.23\right)$.

No.	$x_{H1}$ (m)	$y_{H1}$ (m)	$d_{1}$ (mm)	$x_{H2}$ (m)	$y_{H2}$ (m)	$d_{2}$ (mm)	x (m)	y (m)	z (m)	$x_{err}$	$y_{err}$	$z_{err}$
1	1.40	9.19	20,049	1.41	8.67	20,600	1.33	8.71	−2.97	2.05%	−5.66%	−8.03%
2	1.40	9.17	20,128	1.41	8.41	20,889	1.30	8.51	−3.45	−0.01%	−6.97%	6.68%
3	1.39	9.10	20,180	1.40	8.38	20,915	1.31	8.58	−3.08	−0.77%	−5.75%	−4.73%
4	1.40	8.88	20,390	1.41	8.43	20,863	1.30	8.24	−3.35	−0.08%	−7.32%	3.81%
